# Sphingosine 1-Phosphate Lyase in the Developing and Injured Nervous System: a Dichotomy?

**DOI:** 10.1007/s12035-023-03524-3

**Published:** 2023-07-28

**Authors:** Junhua Xiao

**Affiliations:** Department of Health Sciences and Biostatistics, School of Health Sciences, Swinburne University of Technology, John Street, Hawthorn, VIC 3022 Australia

**Keywords:** Sphingosine 1-phosphate lyase, Sphingosine 1-phosphate, Multiple sclerosis, Glial cells, Microglia, Astrocyte, Neuron

## Abstract

Sphingosine 1-phosphate lyase (SPL) is the terminal enzyme that controls the degradation of the bioactive lipid sphingosine 1-phosphate (S1P) within an interconnected sphingolipid metabolic network. The unique metabolic position of SPL in maintaining S1P levels implies SPL could be an emerging new therapeutic target. Over the past decade, an evolving effort has been made to unravel the role of SPL in the nervous system; however, to what extent SPL influences the developing and mature nervous system through altering S1P biosynthesis remains opaque. While congenital SPL deletion is associated with deficits in the developing nervous system, the loss of SPL activity in adults appears to be neuroprotective in acquired neurological disorders. The controversial findings concerning SPL’s role in the nervous system are further constrained by the current genetic and pharmacological tools. This review attempts to focus on the multi-faceted nature of SPL function in the mammalian nervous systems, implying its dichotomy in the developing and adult central nervous system (CNS). This article also highlights SPL is emerging as a therapeutic molecule that can be selectively targeted to modulate S1P for the treatment of acquired neurodegenerative diseases, raising new questions for future investigation. The development of cell-specific inducible conditional SPL mutants and selective pharmacological tools will allow the precise understanding of SPL’s function in the adult CNS, which will aid the development of a new strategy focusing on S1P-based therapies for neuroprotection.

## Introduction

Sphingosine 1-phosphate (S1P), a signalling lipid of the sphingolipid family, inhibits cell apoptosis and promotes DNA synthesis, cell proliferation, and migration [1–5]. In addition to its well-established roles such as cardioprotective action [6] and anti-inflammation [3, 4], evolving evidence suggests S1P also plays a role in regulating the peripheral (PNS) and central (CNS) nervous systems [7]. During normal brain development, S1P is required for the survival and differentiation of neurons and glial cells [8, 9] and the maintenance of the blood–brain-barrier homeostasis [1, 10]. Disrupted S1P signalling and altered expression level has been implicated in a wide range of diseases including autoimmune diseases such as rheumatoid arthritis [4, 11] and neurodegenerative diseases such as multiple sclerosis (MS) [12], Alzheimer’s disease (AD) [13], Parkinson’s disease, and Huntington’s disease [1, 4, 14]. In MS, the normal level of S1P metabolism and signalling is disrupted, as evident by a significantly lower level of S1P in human MS lesions compared to normal appearing white matter [8]. Similar in AD, there is a global deregulation of S1P signalling or a reduction of S1P synthetic enzymes [15, 16]. Collectively, these findings highlight the importance of S1P in the pathological progression of neurodegenerative diseases such as MS and AD. Therefore, a strategy that can protect against the disruption of S1P signalling or prevent the loss of S1P protein will be highly desirable in clinical translation.

**Fig. 1:**
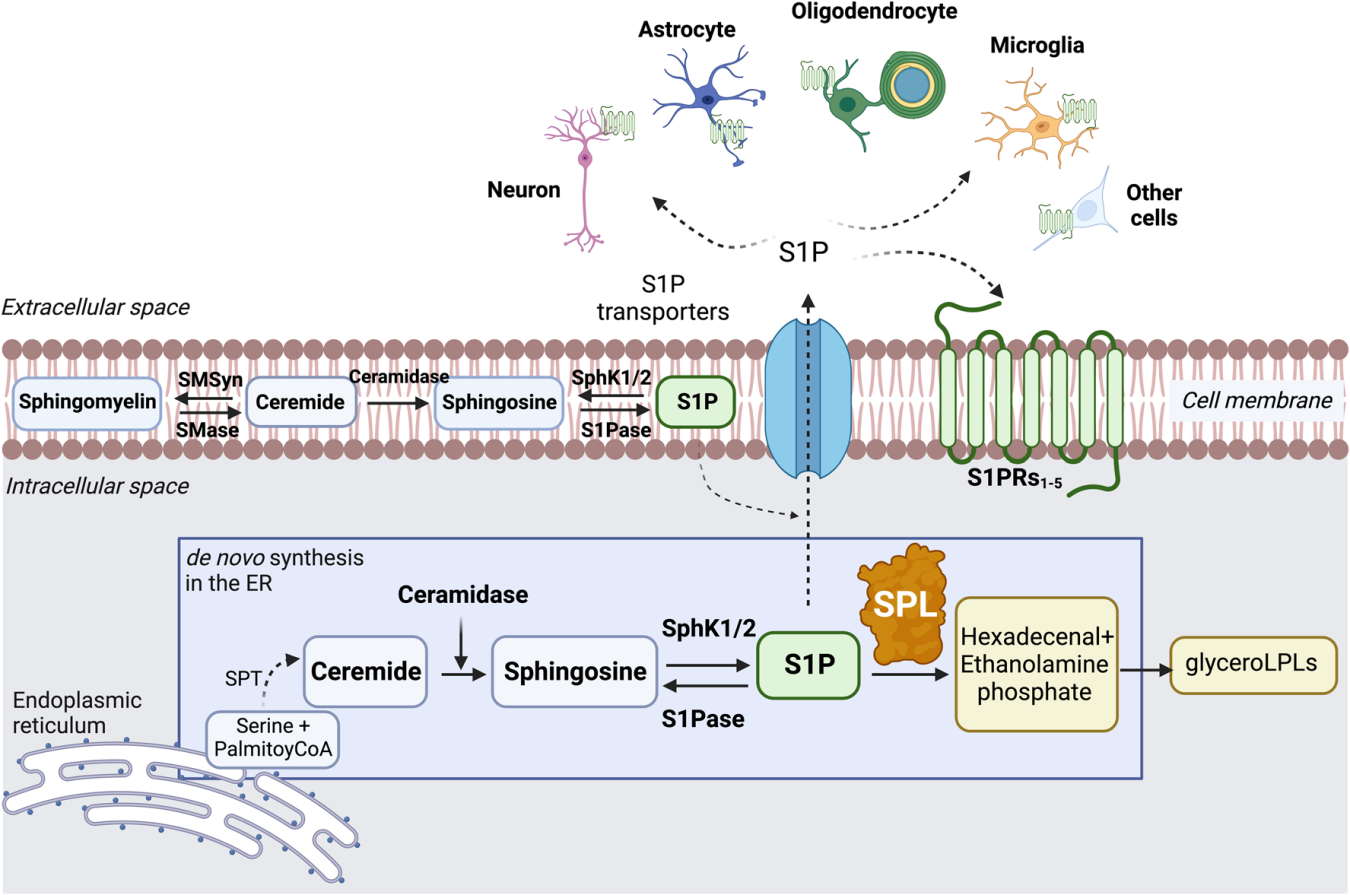
Schematic showing sphingosine-1-phosphate lyase in the catalytic cycle of sphingosine-1-phosphate signalling. Schematic diagram showing that sphingosine 1-phosphate lyase (SPL) controls sphingosine-1-phosphate (S1P) pools available for both intracellular and extracellular signalling. The S1P can be synthesized in the endoplasmic reticulum (ER, de novo synthesis pathway) and at the plasma membrane (salvage synthesis pathway). Intracellular or plasma membrane ceramide can be subsequently metabolized by ceramidase to generate sphingosine which in turn produces S1P. The level of endogenous S1P is kept under control through equilibrium between its synthesis governed by sphingosine kinases 1 and 2 (SphK1/SphK2), and its degradation by SPL. SPL catalyzes the cleavage of S1P, resulting in the formation of hexadecenal and ethanolamine phosphate, which can subsequently enter the glycerolysophospholipids (glyceroLPLs) synthetic pathway. S1P can also be reverted to sphingosine by sphingosine-1-phosphate phosphatase (S1Pase). Figure prepared in Biorender.com with publication license

S1P exerts its biological roles via signalling to a family of five G-protein-coupled S1P receptors 1–5 (S1PR_1-5_) [17]. The amount of S1P available for intracellular and extracellular signalling is kept under control by its synthetic (sphingosine kinases) and degradative (sphingosine 1-phosphate lyase, SPL) enzymes within an interconnected sphingolipid metabolic network (Fig. 1). Significant effort has been made to identify compounds that can modulate S1P signalling through targeting the G-protein-coupled S1P receptors (S1PRs) [18], with the most successful one being fingolimod, a partial agonist of S1P that modulates its receptors _1, 3, 4, 5_ [19]. Despite the clinical benefits in treating patients with relapse-remitting MS (RRMS), fingolimod is associated with off-target side effects such as the risk of stroke and cardiac arrhythmias [18]. Fingolimod and derivatives are non-selective S1PR modulators that possess dual agonism and antagonism properties because of the S1P receptors internalization [20–22]. Interestingly, despite their reported therapeutic effectiveness in neurodegenerative diseases such as MS, targeting S1P signalling cascades via its downstream receptors displayed adverse phenotypes that were not observed in SPL knockout mice in which S1P expression was elevated. For example, elevating S1P levels through genetically ablating SPL expression or pharmacologically inhibiting its activity have both demonstrated a protective effect against myocardium infarction [23], and cardiovascular protection that could not be achieved through modulating S1P-S1PR signalling [6]. It is important to note that these differential effects are almost certainly attributable to the complexity of S1P signalling and the interconnected nature of production, mediated through more than one receptor and one synthetic pathway. The molecular mechanisms by which S1P exerts its biological effects have been challenging to identify [24]. Increasing data on the actual receptor mechanisms of FTY720 suggests while fingolimod is considered a partial analogue, it does not act in the same manner as S1P. Fingolimod-P can bind with S1PR_1,3,4,5_. While both S1P and fingolimod-P induce S1PR_1_ internalization, S1P induces internalization of S1PR_1_ and endosomal recycling to the cell surface, whereas fingolimod-P induces irreversible receptor internalization and degradation, particularly S1PR_1_ [3]. Therefore, these findings collectively indicate that current pharmacological approaches that modulate S1P signalling do not fully recapitulate the biological function of endogenous S1P, arguing the need to search for a new strategy that could selectively target S1P function for the benefit of therapeutic intervention.

SPL holds a unique position in the control of endogenous S1P expression by governing its degradation (Fig. 1). SPL catalyses the irreversible breakdown of S1P, hence acting as the gatekeeper of endogenous S1P expression. S1P level is usually low in lymphoid tissues and high in blood [3, 25]. This normal S1P gradient is required for the egress of T cells from the lymphoid tissues [3]. Inhibition of SPL activity has been shown to restore S1P level in tissues and disrupts the S1P gradient between lymphoid tissues and blood, thus suppressing T cells trafficking to blood and subsequently into the CNS [25]. In this context, SPL could represent a new target that can be modulated to effectively control S1P levels and downstream signalling for therapeutic intervention in immunity. In the nervous system, SPL is present in both neurons and glial cells [7]. While significant effort has been made to unravel the role of SPL in the nervous system including S1P biosynthesis over the past decade, to what extent SPL regulates CNS function via modulating S1P is largely controversial. This review will focus on discussing recent findings that concern SPL function in the mammalian nervous system, with the aim to decipher its roles between the developing and mature nervous systems, and between the congenital and acquired conditions.

## The Complexity of S1P Biosynthesis and Signalling

The S1P can be synthesized via two subcellular pathways: de novo synthesis in the endoplasmic reticulum (ER) and subsequent salvage synthesis at the plasma membrane [7, 26] (Fig. 1). De novo sphingolipid synthesis in the ER is initiated by serine palmitoyltransferase (SPT). Subsequent enzyme reactions in the ER produce ceramide, which is then modified to generate plasma membrane sphingolipids such as sphingomyelin [26]. Intracellular ceramide can be subsequently metabolized by ceramidase to generate sphingosine. At the plasma membrane, the synthesis of S1P via sphingoid base salvage and recycling begins when ceramide is formed by means of a group of sphingomyelinases (SMases). Hence, ceramide is a critical intermediate molecule in the production of bioactive active lipids including sphingosine and S1P. The sphingolipids are interconvertible as sphingosine and ceramide can be interconverted by ceramidase, and sphingosine is reversibly converted into S1P through phosphorylation by SphK (Fig. 1). SPL degrades S1P to form (2E)-hexadecenal and ethanolamine phosphate (Fig. 1), thereby permanently removing S1P from the sphingolipid pool [4, 27]. The amount of bioactive S1P available for both intracellular and extracellular signalling is kept under control through equilibrium between its synthesis governed by sphingosine kinases 1 and 2 (SphK1/2) and degradation by SPL within an interconnected sphingolipid metabolic network (Fig. 1). While SphK1/2 regulates the biosynthesis of S1P, the upregulation of sphingosine kinases does not always increase S1P levels such as in cancer cells, suggesting that regulating sphingosine kinase activity may not lead to a unified outcome of S1P levels [28]. On the other hand, SPL irreversible catalyzes the cleavage of S1P, resulting in the formation of hexadecenal and ethanolamine phosphate. S1P can also be reversibly reverted back to sphingosine by sphingosine-1-phosphate phosphatase (S1Pase). These collectively demonstrate the interconnected nature of S1P synthetic pathways.

S1P exerts its biological roles via signalling to a family of five G-protein-coupled S1P receptors 1–5 (S1PR_1-5_) [17]. Before binding to its cognate receptors, S1P is released from cells in the extracellular milieu through specific transporters [spinster homolog 2 (Spns2) or ABC transporters], allowing signal transduction in autocrine, paracrine, or endocrine fashions. S1PR_1-5_ are widely and variably distributed across different cell types [17, 29, 30]. These receptors are not only present in the cell plasma membrane but also intracellularly including the ER and the cell nucleus [17]. Hence, S1P acts both as an extracellular and intracellular mediator [2]. When acting as an extracellular mediator, S1P effects are dependent on the type and expression levels of the different S1PRs. Different S1PRs are coupled to various G-proteins, subsequently leading to the activation of multiple intracellular signalling pathways such as Ras/ERK, PLC/Ca2 + , P13K/Akt, and Rho/PTEN cascades [17]. Intracellularly, S1P signalling can be dependent or independent of extracellular S1PRs through utilizing different mechanisms [31, 32]. When acting as an intracellular mediator, it has been demonstrated that S1P can function through different cellular compartments, such as the ER and nucleus, inducing histone acetylation [33] and various transcriptional pathways such as NF-κB and FOXO3a [32, 34]. Thus, depending on the form and complement of receptors expressed, S1P can exert different influences and regulate distinct signal transduction pathways in a cell type- and context-specific manner. These further demonstrate the complexity of S1P signalling in addition to its synthetic pathways within an interconnected network, presenting a challenge to selectively modulate S1P synthesis and subsequent signalling.

## SPL: a Gatekeeper of S1P Expression and Lip Metabolic Flow

While the biosynthesis of S1P takes place through two pathways and by means of two enzymatic reactions [5, 7], the gateway that S1P exits the metabolic flow is controlled by one single enzyme, namely SPL. SPL is a member of the PLP (pyridoxal 5′-phosphate)-dependent enzymes superfamily. It is an integral membrane protein, which comprises an N-terminal endoplasmatic lumenal domain, a transmembrane segment, and a soluble PLP-binding domain, the pyridoxal-dependent decarboxylase conserved domain (PDCD). Membrane topology analysis indicates the N-terminus of SPL is located in the lumen of the ER, whereas the large hydrophilic domain containing the active site is located in the cytosol [35]. Human genetic study suggests a shorter (truncated) isoform of SPL exists due to mutations (located before the starting code), resulting in the expression of a shorter transcript (SPL2) containing the functional PDCD domain but without the N-terminal and transmembrane domains [36]. PDCD is the functional domain responsible for PLP binding and enzymatic activity [35]. It not only contains a lysine residue (K353) that acts an active site forming an internal Schiff base with PLP but also well-characterized residues (e.g., Ala388 and Phe545) involved in catalytic activity. The recent crystallization of a eukaryotic SPL [37] together with genetic studies [36, 38] identify that the PDCD domain is evolutionarily conserved from *C. elegans* to humans, providing the molecular basis for SPL-based drug design that targets its catalytic activity. In this context, approaches that can inhibit or activate the catalytic residues within SPL could alter its enzymatic activity that degrades S1P and subsequently alter the de novo production of S1P.

**Fig. 2:**
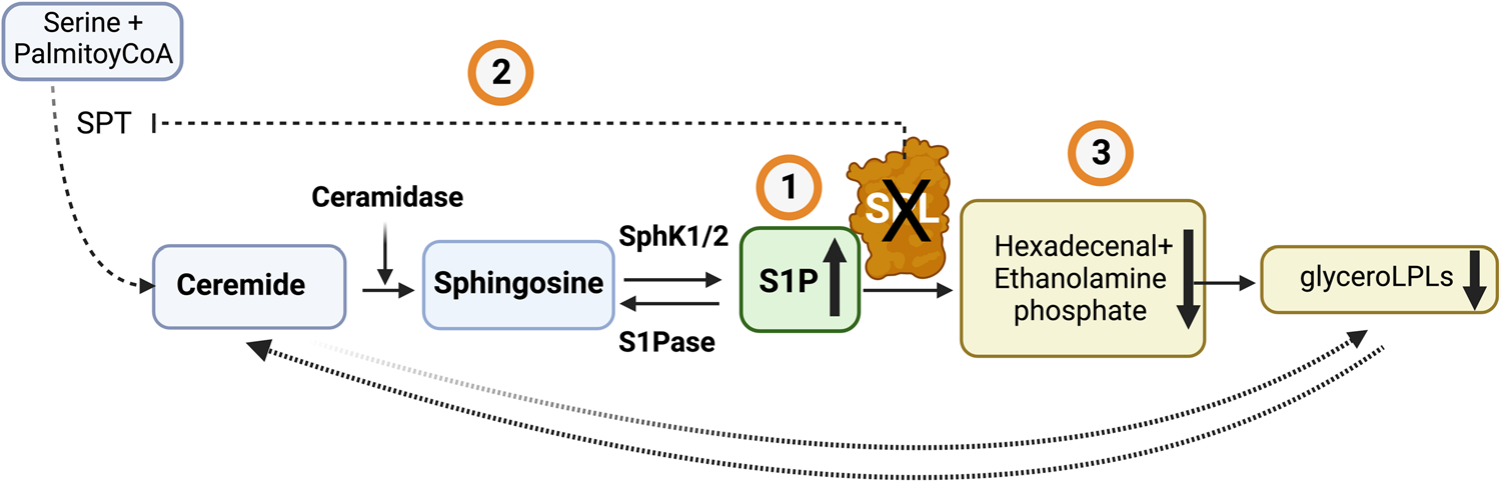
Schematic showing the possible consequences of sphingosine-1-phosphate lyase inhibition. The loss of SPL expression or activity results in not only an overall increase in S1P levels but also a potential change in other sphingolipids as well as glycerolysophospholipids (glyceroLPLs). 1. SPL irreversibly degrades S1P hence the loss of SPL typically leads to an increase in S1P levels. 2. The loss of SPL could reduce de novo biosynthesis of sphingolipids via increasing the recycling of sphingoid bases. 3. SPL facilitates hexadecenal and phosphoethanolamine to enter the glyceroLPLs synthetic pathway. Hence, the loss of SPL could indirectly reduce glyceroLPLs. Production. Figure prepared in Biorender.com with publication license

Being the final enzyme in the degradative pathway of the sphingolipid family, SPL is placed in a unique position to not only control endogenous S1P levels but also regulate the metabolic flow of signalling lipids including sphingosine, sphingomyelin, and ceramide [39]. SPL serves as the primary regulator of cellular and tissue S1P levels [40, 41] hence controls the S1P pool available for intracellular and extracellular signalling through S1PRs (Fig. 1). Moreover, SPL represents the only exit point for sphingolipid intermediates and their flow into phospholipid metabolism through converting S1P to metabolites such as hexadecenal used in phospholipid biosynthesis, hence controlling over the entire sphingolipid metabolic pathway. Therefore, the loss of SPL results in not only an overall increase in S1P but also a potential change in other sphingolipid family members including sphingosine, ceramide, and sphingomyelin [40] (Fig. 2). Interestingly, the loss of SPL such as in neurons was found to increase the recycling of sphingoid bases via ER-resident proteins such as Orm1/3 [42], resulting in a considerable reduction of de novo sphingolipid biosynthesis [43]. This finding suggests that SPL deletion could potentially regulate the internal feedback between the final step in sphingolipid degradation and biosynthesis, a process essential to prevent the loss of synaptic plasticity, neuronal death, and neurodegeneration [Alessenko, 2020 #9, ^44^]. In addition, following S1P degradation, SPL facilitates hexadecenal and phosphoethanolamine to enter the glycerolysophospholipids (glyceroLPLs) synthetic pathway by the actions of the fatty aldehyde dehydrogenase (FALDH) and cytidine diphosphate (CDP)‐ethanolamine (CTP) [45], leading to the production of glyceroLPLs, which display multiple roles in neurogenesis, vascular development, and immunity [46]. Notably, the change of biologically active lipid mediators following SPL modulation occurs in a context-dependent manner [47]. Therefore, this interconnected nature of sphingolipid metabolism indicates that modulating SPL activity could influence not only the amount of S1P available for intracellular and extracellular signalling but also the sphingolipid homeostasis as well as other bioactive lipid mediators such as glyceroLPLs (Fig. 2). Considering the unique position of SPL in the sphingolipid metabolic pathways together with a promising therapeutic potential of S1P, understanding how SPL activity, by implication S1P substrate modulation, influences the nervous system function is crucial.

## SPL Expression in the Mammalian Nervous System

Since Stoffel et al. first described the primary feature of SPL catalytic function approximately 50 years ago [48], there is still scarce data about its cell-type dependent function. Our understanding of SPL’s cellular and subcellular expression profile is still limited. SPL is a microsomal enzyme ubiquitously present in mammalian tissues with the expression of Sgpl1, the gene that encodes full size mammalian SPL, being highly conserved from yeast to humans [38, 39, 49]. In mammals, SPL is expressed in most cells except for blood erythrocytes and platelets, although the relative amount of SPL varies considerably depending upon the organ, tissue, and cell type [35, 50]. Subcellularly, SPL is enriched within the ER [35].

Analysis of the b-galactosidase SPL reporter mouse reveals that the mammalian SPL is expressed in multiple systems, ranging from the immune system, and the nervous system, through to the reproductive and endocrine organs [50]. This broad spectrum of SPL expression profile not only indicates its integral role in regulating mammalian physiology and homeostasis but also suggests SPL has many undiscovered functions apart from innate and adaptive immunity, a heavily focused area of current SPL research.

Indeed, SPL is present in both the PNS and CNS [35, 50]. Analysis of mouse embryos reveals that SPL is expressed in the developing brain and neural tube as well as peripheral nerve tissues [51], suggesting it is involved in nervous system development. SPL mRNA expression in mouse tissues appears to be developmentally regulated, with a transiently high expression observed from embryonic day 5.5 (E5.5) to E7.5, a critical period to mouse embryogenesis including neurogenesis [35]. These findings are consistent with congenital abnormalities observed in SPL mouse mutants and humans with SPL mutations, the latter of which is associated with congenital brain malformation [52]. Overall, the expression profile of SPL in the developing CNS together with the associated genetic phenotypes suggest that endogenous SPL expression is required for the normal CNS structure and function.

In the mouse brain, SPL expression is enriched in several CNS regions including the cerebrum, cerebellum, spinal cord, choroid plexus, and the olfactory bulb [50, 51]. This is consistent with Human Protein Atlas data demonstrating that Sgpl1 is expressed throughout the grey and white matter of the human brain (https://www.proteinatlas.org/ENSG00000166224-SGPL1/brain). At a cellular level, Barry’s transcriptome analysis of nerve cells in the developing mouse brain [53] together with the Human Protein Atlas demonstrate that SPL gene (Sgpl1) and protein are expressed in both neurons and glial cells including oligodendrocyte precursor cells (OPCs), mature oligodendrocytes, astrocytes, and microglia, with relatively high expression being detected in astrocytes and OPCs. Moreover, the microarray data from Cahoy et al. suggests that SPL expression within murine astrocytes declines during early development from postnatal day 7 (P7) to P17 but remains steady in neurons during the same period [53]. Although the data is limited and requires further analysis, it sheds light on the potential role that SPL plays in glial cell function during early postnatal development. However, whether SPL expression across different CNS regions and neural cell types remains steady or changes from birth to adulthood remains unknown. Notably, various studies have demonstrated a diverse profile of S1PR expression in the CNS, with neurons expressing S1PR_1 3 5_, astrocytes expressing S1PR_1 3 4_, microglia expressing S1PR_4_, and oligodendrocytes expressing S1PR_5_ [24, 54]. The diverse cellular profile of S1PR implies that modulating S1P such as regulating SPL activity is likely to exert multi-faceted effects in the CNS.

## SPL Is Required for Normal Nervous System Development

The expression profile of SPL in the developing brain and neural tube suggests that endogenous SPL expression is involved in the normal CNS structure and function. Interestingly, mutants in various model organisms such as *Caenorhabditis elegans* and *Drosophila melanogaster* have revealed developmental deficits including growth and reproductive defects [55, 56]. Silencing SPL expression in Drosophila neurons triggers developmental defects in peripheral neurons and neuromuscular junction [57]. In mammals, indirect evidence from knockout mice that lack the ability to produce S1P or signal through S1PR are embryonic lethal and display interrupted angiogenesis and neurogenesis, increased neuronal apoptosis, and reduced cell proliferation in the telencephalon [58], indicating that S1P is involved in promoting angiogenesis and neurogenesis during development and that modulating SPL activity could influence neural development. In support of this, patients harboring mutations in Sgpl1 often present with developmental neurological pathologies. Recently, a series of SPL mouse mutants have been generated and these findings collectively shed new light on the roles that SPL plays in the mammalian nervous system in a developmental context.

### SPL in Mouse Brain Development

**Fig. 3:**
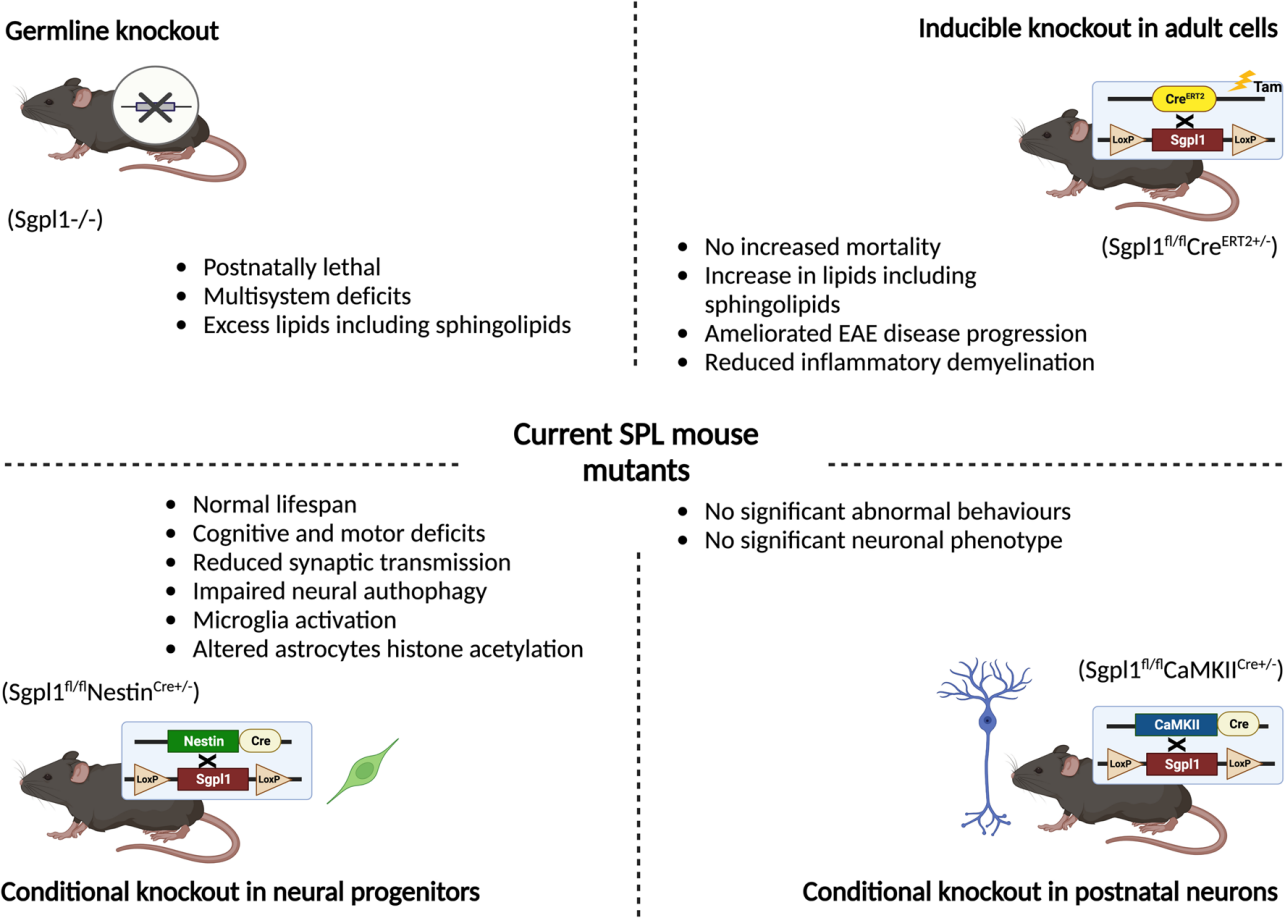
Schematic summarizing the phenotype of current SPL mouse mutants. Current phenotypic analysis of SPL mouse mutants. Germline deletion of SPL (SPL^−/−^) is postnatally lethal and leads to developmental deficits in multiple systems [40, 60]. Developmental conditional ablation of SPL in neural progenitor cells shows deficits in cognitive and motor skills accompanied by reduced synaptic transmission [62, 64] and altered glial cells function [54, 65, 67], while the loss of SPL in postnatal neurons [62] is less penetrant in detectable phenotypes. In contrast, adopting a Tamoxifen (Tam)-inducible knockout strategy, the loss of SPL function in adult cells is neuroprotective in the context of acquired neurological disorders such as the Experimental Autoimmune Encephalomyelitis (EAE) model of multiple sclerosis [70]. Figure prepared in Biorender.com with publication license

Over the past 10 years, a series of SPL mouse mutants including global and conditional knockouts have been established (Fig. 3). Increased S1P accumulation and lipids including sphingolipids are common biochemical outcomes observed in most SPL mouse mutants; however, the nervous system phenotypes vary a great deal (Fig. 3). The global germline deletion of SPL in mice leads to a pronounced increase in S1P production [59] accompanied by developmental defects. While SPL null (Sgpl1^−/−^) mice are born normally, they are postnatally lethal [50, 60] although a few can survive into late postnatal weeks [61]. Interestingly, mice with the constitutive deletion of SPL in neural progenitor cells (Sgpl^fl/fl^ Nestin Cre^+/−^) or postnatal neurons (Sgpl^fl/fl^ CaMKII Cre^+/−^) have normal lifespan compared to their wildtype littermate controls [62] (Fig. 3). In support of this finding, mice with blocked S1P signalling in CNS cells display no survival issues and normal CNS structures [63], implying that the expression of SPL in CNS residential cells does not dictate survival. Interestingly, SPL depletion in neural progenitor cells (Sgpl^fl/fl^ Nestin Cre^+/−^) during mouse embryonic development displays significantly reduced synaptic transmission accompanied by prolonged deficits in skills of spatial learning, memory, and motor coordination [62, 64] (Fig. 3). A subsequent analysis of the mouse mutant demonstrates that constitutively deleting SPL in neural progenitor cells is associated with hyperphosphorylated tau [65]. These findings collectively indicate that the loss of SPL (by implication S1P accumulation) regulates the presynaptic architecture and neuronal autophagy in vivo. Indeed, neuroanatomical studies reveal that cerebellum neurons with abundant S1P-lyase expression degenerate first in SPL-deficient mice [61]. This is also supported in vitro, in which S1P accumulation following the loss of SPL leads to neurotoxicity in postmitotic central neurons [61]. Early studies demonstrate that the amount of ceramide remains intact in SPL-deficient neurons [43, 66] and that the loss of SPL in neurons readjusts the balance between de novo sphingolipid biosynthesis and recycling to maintain (glyco)sphingolipid homeostasis [43]. Collectively, current in vitro and in vivo findings suggest that SPL depletion in the developing CNS is neurotoxic, a process involving the regulation of sphingolipid biosynthesis such as the S1P-ceramide balance [5].

The impact of SPL in the CNS extends beyond neurons toward glial cells [5]. In SGPL1^fl/fl^ Nestin^Cre^ mice, microglia were found activated as evidenced by de-ramified morphology accompanied by the increased expression of microglial activation markers and proinflammatory cytokines [67]. In addition to microglia, increased histone acetylation was found in SGPL1-deficient astrocytes, but not neurons, derived from SGPL1^fl/fl^ Nestin^Cre^ mice [65], indicating glial cells could be the primary cell type that regulates the central effects of S1P in the CNS. This was supported by a genetic study that ablating SPL in the postnatal forebrain-specific neurons (Sgpl^fl/fl^ CaMKIICre^+/−^) resulted in an unremarkable phenotype in the brain [62]. The aforementioned CNS phenotypes observed in the neural progenitor deletion of SPL (Sgpl^fl/fl^ NestinCre^+/−^) were not detectable in neuronal depletion of SPL (Sgpl^fl/fl^ CaMKIICre^+/−^) in vivo. Notably, only a mild change in both SPL and S1P expression was detected in limited CNS regions of Sgpl^fl/fl^ CaMKIICre^+/−^ mice [62]. Hence, the distinct phenotypes between the two CNS cell-specific SPL mouse mutants not only suggest a difference in the Cre recombination efficacy between the two lines but also importantly indicate that the key cellular source of SPL in the CNS could lie mostly with glial cells rather than neurons.

While S1P metabolism plays a role in synaptic plasticity [7], the precise role that SPL plays in the developing CNS via degrading S1P remains inconclusive. It remains interesting to understand how S1P accumulation, following SPL depletion in CNS cells, alters the function of neurons or other glial cells. SPL deletion in the neural progenitors increases SPL levels within the intra- and extracellular environment [67]. In particular, astrocytic but not microglial S1P mediates the secretion of proinflammatory factor interleukin-6 (IL-6) in cultured microglial cells [67], priming their activity to an inflammatory insult. Using a pharmacological approach, it has been further demonstrated that S1PR_2_ expressed by the microglia is a main mediator of impaired autophagy and proinflammatory effects following SPL depletion in neural progenitors [67]. In addition, SPL deficiency in astrocytes, and hence the accumulation of its substrate S1P, impairs glucose degradation and autophagy in cultured astrocytes, presumably via S1PR_2 4_ [54]. Therefore, the loss of SPL can alter the function of both neurons and glial cells such as microglia and astrocytes, both of which are involved in synaptic plasticity and cognition [68, 69]. Therefore, it will be interesting to investigate whether the cognitive deficits observed in the Sgpl^fl/fl^ NestinCre^+/−^ mouse [62, 64] are due to a direct effect on neural damage or secondary to altered glial cell function [67]. Considering the close association between S1P-induced neuroinflammation and autophagy [5, 54], future investigation is required to determine how the glial cells-expressing SPL regulates S1P function pertinent to neuron-glia interaction. In addition to regulating the terminal production of S1P, the amount of SPL also plays a role in de novo sphingolipid biosynthesis such as the synthesis of sphingosine as well as sphingomyelin [40, 43], potentially through modifying the recycling of sphingoid bases [42]. However, currently published genetic tools have yet to be able to dissect to what extent SPL regulates the nervous system function through governing sphingolipid biosynthesis.

Nevertheless, these mouse genetic tools have provided insightful evidence that SPL is essential to the developing nervous system [40, 62, 65] and that SPL-mediated S1P metabolism regulates the function of both neurons [40, 62] and glial cells [54, 65, 67]. Findings of these SPL mouse mutants also indicate a clear stage-specific and cell-type-specific role of SPL in the CNS. Given the increasingly important roles that glial cells play in neuroplasticity and neurological diseases, a more complete understanding of SPL in glial cells such as microglia and astrocytes will provide new insights toward developing new therapeutics that target S1P. Despite the exciting genetic findings, however, there is still a lack of cell-specific inducible SPL mutant mice, which represents a clear gap to dissect the role that SPL plays in the adult nervous system, pertinent to neurodegenerative diseases in which the S1P level is interrupted.

### SPL in Human Brain Development

The human SPL gene (Sgpl1) shares 84% identity with mouse SGPL1 [38, 49]. The aforementioned phenotypes of SPL mouse mutants are overall in line with human genetic studies [56, 60]. Neurological abnormalities have been reported in approximately 50% of the clinical cases reporting SPL insufficiency [52]. These neurological manifestations include macrocephaly, microcephaly, hypotonia, cranial nerve palsies, sensorineural hearing loss, seizures, peripheral neuropathy, ataxia, and developmental delay [52]. Progressive decline in motor and cognitive skills, as well as the impaired acquisition of new skills, are described in some patients who carry SPL mutations during early development [60], similar to the impaired cognitive and motor skills observed in the SPL mouse mutant (Sgpl^fl/fl^ NestinCre^+/−^) [62, 64]. MRI scan of infants with SPL insufficiency indicates cerebral and cerebellar cortical lesions in the grey matter such as the basal ganglia and the white matter such as the corpus callosum [52], These findings collectively suggest that the congenital loss of SPL impedes normal brain development in human, consistent with the findings observed in SPL mouse mutants.

## SPL in the Adult Nervous System: Implications for Acquired Neurodegenerative Diseases

Still little is known about the role of SPL in the adult nervous system function. It is also unclear whether and/or to what extent SPL expression changes during development and into adulthood. An inducible genetic approach enables a partial deletion of SPL in the adult mouse [70]. The adult SPL mouse mutant that expresses approximately half amount of SPL displays a normal life span compared to the littermate control mouse [70]. However, apart from this study, there is little study that provides direct evidence of SPL in the adult CNS. Albeit, modulating S1P level via the synthetic sphingosine kinases (SphK1 and SphK2) implies a potential new role that SPL could also play in the adult CNS via regulating S1P synthesis. Reduced S1P synthesis following SphK2 deletion results in significantly thinner myelin sheath without affecting the number of oligodendrocytes compared to the control [71]. This key finding not only indicates that S1P is required for myelin maintenance in the aging CNS but also raises an important question as to whether targeting S1P synthesis, e.g., SphK2 activation or SPL inhibition, could be a new strategy to enhance myelin formation or maintenance. Despite this finding, no dramatic CNS phenotype has been reported in adult mouse mutants in which SPL expression or S1P signalling is interrupted, indicating that S1P together with SPL may not play a major role in maintaining the adult nervous system, but could be involved in specific cellular events such as myelin formation. That said, in a stressed environment where the nervous system is challenged by an insult, the loss of SPL activity is linked with therapeutic benefits in acquired neurodegenerative diseases such as AD, MS, Parkinson’s disease (PD), and Huntington’s disease (HD) [1, 13].

### SPL in Alzheimer’s Diseases

Growing evidence suggests that SPL could be directly or indirectly involved in the neuropathological development of AD [72]. SPL governs the physiological level of S1P that is known to protect against DNA damage [73], a common mechanism underlying neural cell death in neurodegenerative diseases such as AD [74]. DNA damage induces endogenous SPL expression which subsequently leads to p53- and p38-mediated cell death [75]. While these experiments are conducted in HEK cells and human colon cancer tissues, such findings still shed light on a potential mechanism through which SPL induces cell death in other systems such as the nervous system. Indeed, exogenous S1P has been shown to protect against the death of cultured central neurons [76]. Interestingly, the S1P-dependent neurotoxicity appears to share a similarity with the amyloid β-peptide (Ab) neurotoxicity in AD [15, 16, 61], the latter of which also involves procaspase-12 activation by disruption of ER calcium homeostasis but not by membrane- or mitochondria-targeted apoptotic signals [77, 78]. In the human AD brain, there is an increase in the expression of SPL coupled with a reduction of S1P synthetic enzyme SphK1, by implication low S1P levels, which correlates with amyloid deposits in the entorhinal cortex [15], the gateway to the hippocampus and affected in an early stage of AD. In support of this, the S1P level is reduced in a mouse model of AD, resulting in smaller hippocampus accompanied by neuronal network deficits in vivo [79]. Increased SPL expression disrupts S1P metabolism and reduces its subsequent signalling, which is believed to be associated with the accumulation of Aβ peptides [15, 16]. Likewise, SPL inhibition, by implication S1P restoration, reduces the metabolism of amyloid precursor protein (APP) and the activity of y-secretases [80]. Cellular amyloid protein accumulation, the fundamental reason for AD, is suppressed when SPL is deficient, indicative of neuroprotection. Taken together, these findings highlight a pivotal role that SPL could play in neuroprotection via regulating S1P metabolism, indicating a new therapeutic strategy for the treatment of AD for future investigation.

### SPL in Multiple Sclerosis

SPL also regulates neuroregeneration relating to MS. Inducible ablation of SPL in adult mice ameliorates the disease progression and severity of Experimental Autoimmune Encephalomyelitis (EAE), an animal model of MS for studying inflammatory demyelination [70]. Billich et al. [70] identify that SPL deficiency in adult cells not only suppresses T cell egression but also importantly protects against the extent of inflammatory myelin damage. Notably, however, following the partial depletion of SPL in the adult CNS, the beneficial effect observed in the EAE model is likely driven by peripheral inflammatory suppression as evidenced by the profound reduction of peripheral T cells while the SPL activity in the CNS remains intact [70]. While the beneficial phenotype is peripheral-driven and likely due to a sub-optimal recombination efficiency of the inducible genetic model, the finding of this study raises a key question for future investigation as to whether the CNS-expressing SPL or S1P plays a role in the progression of EAE as well as MS. Nevertheless, this is a milestone finding as it is the first genetic study that has provided both functional and structural evidence demonstrating endogenous SPL is involved in the EAE disease progression and that blocking its activity in adult ameliorates MS-like disease progression and neural pathology. Future research is absolutely required to determine whether blocking SPL activity in adult CNS plays a role in directly promoting neuroprotection in addition to suppressing informatory neurodegeneration. Interestingly, analysis of a toxin (cuprizone)-induced mouse model of CNS demyelination shows that reducing S1P biosynthesis through deleting SphK2 results in a significantly greater loss of mature oligodendrocytes and impairs remyelination compared to the control condition [71], indicating an essential role of endogenous S1P in promoting myelin repair after injury. Similarly, another study from the same laboratory demonstrates that, in a mouse of AD, deleting SphK2 leads to fewer oligodendrocytes and reduces the expression of myelin proteins [79]. Moreover, SPL deficiency not only increases S1P production but also alters the level of other sphingolipids including ceramide and sphingomyelins [40] but in a tissue-specific manner. In the liver, SPL deficiency results in a remarkable elevation of S1P (over 400-fold) and sphingosine (over 40-fold) [40], the increase of which, however, is approximately 10 times less in cerebellar neurons [43, 66]. Moreover, the loss of SPL increases the mass of ceramide and sphingomyelin in the liver [40] but not in central neurons [43, 66], suggesting a tissue-specific consequence in sphingolipids biosynthesis following SPL depletion. Sphingomyelin is an important component of myelin sheath formation. Reduced sphingomyelins expression induces oligodendrocyte death [81]. Likewise, forced expression of sphingomyelins in adult oligodendrocytes not only protects against the extent of demyelination but also promotes remyelination and myelin compaction in the adult CNS [81]. These pre-clinical findings in mice are in line with human studies, although limited. In human MS brains, the S1P level is significantly lower in MS lesions compared to normal appearing white matter [8], indicating that the normal level of S1P metabolism could be disrupted in human MS accompanied by comprised neuroprotection involving S1P signalling [12]. Collectively, these findings together with the genetic studies indicate that the role that SPL plays in the developing and adult nervous system in particular after injury could be largely distinct. When the nervous system is challenged by an insult, SPL inhibition (by implication S1P restoration) could be neural reparative such as remyelination.

The recent studies of human pathology together with pre-clinical animal models of diseases have shed light on the roles of SPL in neuroprotection and that SPL could be a future therapeutic target for MS. Indeed, significant effort has been made to identify compounds that target either SPL or S1P [18, 72] with the most successful one being fingolimod, a partial analogue of S1P and non-selective modulator of S1P receptors_1, 5_ [19]. While modulating S1P signalling via the pharmacological approaches has shown beneficial outcomes on remyelination [18], fingolimod and its derivatives are only partially effective in a proportion of MS patients, primarily relapse-remitting forms of MS. Importantly, the no-selective nature of fingolimod and its derivatives have induced unwanted side effects [18, 82]. In this context, preventing the loss of endogenous S1P through selectively inhibiting SPL could be a new strategy for ameliorating neurodegenerative diseases such as AD and MS for future clinical translation.

## Development of SPL Inhibitors: an Alternative Strategy to Modulate S1P Level

A more precise understanding of both S1P and SPL function in health and disease requires both genetic and pharmacological tools. From a therapeutic perspective, the implication of S1P signalling in a wide range of diseases necessitates the development of specific SPL inhibitors not only for understanding its role in pathological conditions but also for determining its mechanism of action. Indeed, several screen assays have been undertaken to identify the pharmacological tools that target SPL [72, 83–85]. However, only a few inhibitors of SPL have been identified. These include the 2-acetyl-4-(tetrahydroxybutyl) imidazole (THI) [25, 86–88], THI derivatives (LX2931 and A6770) [25, 84, 89], the PLP analogue 4-deoxypyridoxine (DOP) [25], [(4-benzylpthalazin-1-yl)-2-methylpiperazin-1-yl] nicotinonitrile [40, 84], compound 28 [90], and RBM10-8 [91].

THI is a component of caramel color III widely used in the food industry [86]. DOP is a vitamin B6 antagonist [86] that suppresses T cell egression, similar to THI [25]. Inhibiting SPL using THI has been shown to reduce the size of myocardium infarct size, a cardiovascular protective effect similar to SPL deletion [23]. LX2931 is a derivative of THI that has been shown to reduce joint damage in collagen-induced arthritis in mice with a promising safety and efficacy profile in phase I clinical trials which has subsequently undergone phase II clinical trials to treat patients with rheumatoid arthritis [11]. However, while THI and its derivatives were found to reduce SPL activity when administered in vivo [25, 92], the specificity of inhibiting SPL activity is not fully recapitulated via genetic studies in vitro [25]. Compound RBM10-8 is identified as a vinylated analogue of S1P [91]. It is reported to not only behave as an enzyme substrate but also irreversibly inhibit human SPL. Future biological evaluation of RBM10-8 in both in vitro and in vivo models will boost the confidence of this newly identified S1P analogue. Recently, [(4-benzylpthalazin-1-yl)-2-methylpiperazin-1-yl] nicotinonitrile [40] is identified as the first and direct SPL inhibitor through using purified SPL in in vitro assays. One of the derivatives, compound 31, possesses higher competency in suppressing SPL activity [84]. Importantly, the administration of compound 31 in the EAE model of MS significantly suppresses the progression of EAE disease and the extent of symptom severity accompanied by eliminated lymphocyte infiltration in myelin lesions in the EAE spinal cord [84]. This is encouraging as the finding suggests that inhibiting SPL activity could ameliorate disease progression and neurodegeneration in the context of MS, mirroring the EAE phenotype induced in the adult SPL mouse mutant [70]. However, in the study by Weiler et al., compound 31 is administrated 2 days before the EAE disease induction [84], which is an immune-suppressing paradigm but not a therapeutic paradigm. Therefore, the result of this study only demonstrates the immune suppression role of SPL inhibition in the induction of EAE disease; however, whether SPL inhibition plays a therapeutic role in preventing against neurodegeneration in the context of MS is unclear. Therefore, there is a present need to develop new direct SPL inhibitors with proven specificity and therapeutic efficacy in neuroprotection.

## Future Perspectives

SPL is required for maintaining the physiological levels of S1P and other sphingolipid intermediates, contributing to both normal development and injury responses. While S1P plays an essential role in brain development [58, 93], its role in neurodegenerative processes is still under debate [7]. Several key questions need to be addressed to have a more complete understanding of S1P signalling and the therapeutic role of SPL.

### Does the CNS-Expressing SPL Play a Direct Role in Neuroprotection?

A key question yet to be addressed is whether the CNS-expressing SPL plays a direct role in neuroprotection after injury. While recent genetic studies consistently demonstrate that the expression of SPL in the CNS is required for normal nervous system development [62, 64, 65], the role of CNS-expressing SPL in maintaining the adult CNS and importantly regulating neural repair after injury is largely unknown. Genetic studies support the role of CNS-expressing S1P in neuroprotection in the context of neurodegenerative diseases. A global deletion of SphK2 results in fewer myelin-forming oligodendrocytes and a loss of myelin protein expression in animal models of demyelination or AD [71, 79], implying a role of CNS-expressing S1P in neuroprotection after injury. Therefore, in this context, selectively modulating central cell-expressing SPL is likely to restore S1P levels within the CNS after injury, which should, in theory, exert direct beneficial effects upon neuroprotection. However, there are limited genetic studies that have modulated SPL activity selectively in central nerve cells, in particular adult mice. Only one study shows that partial deletion of SPL in adult mice displayed significant protection against EAE disease severity as well as inflammatory demyelination [70]. While this finding was exciting, these beneficial effects are driven by the peripheral-expressing SPL rather than its CNS source [70]. Therefore, it is still unclear whether the expression of SPL in the adult CNS plays a direct role in neuroprotection after acquired injury. Future research is required to address this question by using both genetic and pharmacological tools that can selectively modulate SPL expression or activity in the adult CNS and to test their efficacy in vivo using a therapeutically relevant paradigm.

### Does Modulating SPL Activity Alter S1P Levels Within the CNS?

Currently, there are only a limited number of SPL inhibitors being reported, many of which demonstrate their efficacy in inhibiting SPL activity and elevating S1P but only in the periphery [25, 40, 84, 87, 88, 90]. Systematic administration of the published SPL inhibitors demonstrates their profound biological efficacy in elevating S1P levels in peripheral tissues such as the spleen. These include compound 31, the first director inhibitor of SPL that shows a significant effect in suppressing the progression of EAE [84] and THI and its derivatives [25, 87]. Biologically, these inhibitors share similar patterns in suppressing peripheral inflammation. While this is exciting, none of the published SPL inhibitors has displayed proven efficacy in modulating S1P levels within the CNS. Indeed, whether and to what extent these reported SPL inhibitors exert a direct biological role in the CNS is unknown, understanding of which is critical to dissect their therapeutic implications. With the growing interest in exploring S1P signalling for neuroprotection in neurological diseases, having a valid SPL inhibitor with proven efficacy in elevating S1P levels in the CNS is critical.

### Need More Sophisticated Tools to Study SPL Function

The lack of sophisticated genetic tools (e.g., cell-type-specific inducible SPL mouse mutant) and specific pharmacological tools (e.g., direct SPL inhibitors) have together posed challenges to the precise understanding of SPL function and therapeutic application in the adult CNS. This ultimately limits the development of S1P-based therapeutic intervention toward targeting congenital and acquired neurological conditions. It is important to note that modulating S1P signalling via targeting S1PRs should not be viewed as the same approach as modulating endogenous S1P levels, the latter of which influences not only the signalling downstream of S1P but also the internal feedback to sphingolipid biosynthesis, hence possessing an additional effect on lipid metabolism. For example, fingolimod (FTY720) is a partial analogue of S1P and a non-selective modulator of S1P receptors [19]. S1P can induce the expression of neural trophic factors including the brain-derived neurotrophic factor, leukemia inhibitory factor, and platelet-derived growth factor B in primary human and murine astrocytes to a much greater extent than FTY720 [76]. Moreover, S1P, but not FTY720, protects against excitotoxic neuronal cell death in a primary murine neuron-glia coculture model [76], suggesting that S1P modulators such as FTY720 exert their biological effects through a mechanism different to endogenous S1P and that FTY720 possesses relative inefficacy compared to S1P. Moreover, current S1P-based therapies are non-selective S1PR modulators that possess both agonism and antagonism properties due to S1PR_1-5_ internalization [20–22]. Therefore, the lack of both sophisticated genetic tools and selective pharmacological approaches represents a technical challenge to fully understand the function of SPL in S1P metabolism, ultimately impeding therapeutic development for acquired neurological diseases. Therefore, this is a demonstrated need to develop selective and sophisticated tools for SPL in order to fully interrogate its function.

## Summary

This review highlights the importance of S1P functions in the CNS through deciphering the dynamic role of its degradation enzyme SPL. Constitutive deletion of SPL during early development leads to a series of developmental deficits including the brain. While the precise role that SPL plays in the adult CNS remains inconclusive, blocking SPL activity or restoring S1P after CNS injury could be protective. While this discrepancy between the developing and mature CNS remains largely unclear, the underlying mechanisms through which SPL regulates the nervous system function (e.g., neuronal survival and proliferation) in a healthy or stressed environment are likely to be distinct. Hence, SPL could play a dichotomy in the mammalian nervous system, and finding interpretation is highly context-dependent. The approach to modulate S1P levels, via targeting its synthesis or degradation, could circumvent potential side effects due to receptor internalization that might otherwise occur. The identification of specific SPL inhibitors would advance future studies focusing on investigating SPL function, hence S1P level modulation, in a wide range of experimental systems. Since strategies to modify SPL activity are being explored in both pre-clinical and clinical trials of several conditions, including MS and rheumatoid arthritis, further characterization of SPL inhibition in pre-clinical disease models together with transgenic mouse approaches with a CNS focus will provide new insights into the development of new S1P-based therapies for the therapeutic benefits of neurodegenerative diseases.

## Data Availability

Not applicable.
